# Laboratory-based efficacy evaluation of *Bacillus thuringiensis* var. israelensis and temephos larvicides against larvae of *Anopheles stephensi* in ethiopia

**DOI:** 10.1186/s12936-023-04475-9

**Published:** 2023-02-09

**Authors:** Abebe Teshome, Berhanu Erko, Lemu Golassa, Gedeon Yohannes, Seth R. Irish, Sarah Zohdy, Sisay Dugassa

**Affiliations:** 1 National Malaria Elimination Programme, Ministry of Health Ethiopia, PO Box 1234, Addis Ababa, Ethiopia; 2 Aklilu Lemma Institute of Pathobiology, Addis Ababa University, PO Box 1176, Addis Ababa, Ethiopia; 3 Department of Zoological Sciences, Addis Ababa University, PO Box 1176, Addis Ababa, Ethiopia; 4 Swiss Tropical and Public Health Institute (Swiss TPH), 4123 Allschwil, Switzerland; 5 Centers for Disease Control and Prevention, US President’s Malaria Initiative, Atlanta, GA USA

**Keywords:** *Anopheles stephensi*, Temephos, *Bti*, Susceptibility, Malaria, Vector control

## Abstract

**Background:**

Malaria, transmitted by the bite of infective female *Anopheles* mosquitoes, remains a global public health problem. The presence of an invasive *Anopheles stephensi,* capable of transmitting *Plasmodium vivax* and *Plasmodium falciparum* parasites was first reported in Ethiopia in 2016. The ecology of *An. stephensi* is different from that of *Anopheles arabiensis*, the primary Ethiopian malaria vector, and this suggests that alternative control strategies may be necessary. Larviciding may be an effective alternative strategy, but there is limited information on the susceptibility of Ethiopian *An. stephensi* to common larvicides. This study aimed to evaluate the efficacy of temephos and *Bacillus thuringiensis* var. israelensis (*Bti*) larvicides against larvae of invasive *An. stephensi*.

**Methods:**

The diagnostic doses of two larvicides, temephos (0.25 ml/l) and *Bti* (0.05 mg/l) were tested in the laboratory against the immature stages (late third to early fourth stages larvae) of *An. stephensi* collected from the field and reared in a bio-secure insectary. Larvae were collected from two sites (Haro Adi and Awash Subuh Kilo). For each site, three hundred larvae were tested against each insecticide (as well as an untreated control), in batches of 25. The data from all replicates were pooled and descriptive statistics prepared.

**Results:**

The mortality of larvae exposed to temephos was 100% for both sites. Mortality to *Bti* was 99.7% at Awash and 100% at Haro Adi site.

**Conclusions:**

Larvae of *An. stephensi* are susceptible to temephos and *Bti* larvicides suggesting that larviciding with these insecticides through vector control programmes may be effective against *An. stephensi* in these localities.

**Supplementary Information:**

The online version contains supplementary material available at 10.1186/s12936-023-04475-9.

## Background

Malaria is a global public health problem that mainly affects tropical countries [1]. It is transmitted by the bite of infective female *Anopheles* mosquito species. Globally, there are some 3530 species of mosquitoes under 43 genera in the family Culicidae, which are further divided into the subfamilies of *Culicinae, Anophelinae* and *Toxorhynchitinae* [2]. Of these, malaria vectors belong to the genus *Anopheles* [2].

In Ethiopia, *Anopheles arabiensis* is the main malaria vector while *Anopheles pharoensis, Anopheles funestus* and *Anopheles nili* are secondary vectors [3]. The recently reported invasive species, *Anopheles stephensi* in the country has exhibited the potential of transmitting *Plasmodium falciparum* and *Plasmodium vivax* [4, 7]*.* The species has also been reported from other countries in the Horn of Africa including Djibouti (2012), Sudan (2016), and Somalia (2019), raising concern about appropriate vector control strategies to target this invasive species [4, 5].

Unlike other malaria vectors, *An. stephensi* is also considered an urban and peri-urban adapted malaria vector, which breeds in man-made habitats such as overhead tanks, ditches and canals [5–7]. *Anopheles stephensi* feeds on both humans and animals, with a preference for the latter, and it exhibits more outdoor feeding [4]. *Anopheles stephensi* in Ethiopia is resistant to most insecticides used in current malaria vector control tools, insecticide treated bed-nets (ITN) and indoor residual spraying (IRS) [7, 9], so larviciding might be an effective control method [7].

Larval source management (LSM) is one of the oldest and primary strategies used throughout the world to control malaria targeting the immature stages of the mosquito vectors in their aquatic habitats; however it has been less commonly used in African countries following the introduction of indoor residual spraying (IRS) in the 1950s and long-lasting insecticide impregnated nets (LLINs) in the 1990s [10, 11]. Organophosphates larvicides such as temephos and pirimiphos-methyl interfere with the nervous system of the immature larval stages, whereas naturally occurring microbes such as *Bacillus thuringiensis* var. israelensis (*Bti*) and *Bacillus sphaericus* (*Bs*) kill larvae with their toxins when ingested [10, 11].

In Ethiopia, concerted efforts have been made in the fight against malaria since the 1950s. The intervention strategies have included early diagnosis and prompt treatment of cases, IRS, prevention and control of epidemics, and recently, scale-up of LLINs and LSM through larviciding and environmental management at small scale, where breeding sites are few, findable and manageable [3, 11]. However, resistance to insecticides used in vector control by *An. stephensi* has been reported from within the country [9] and other places [5]. Therefore, in order to tailor the local strategies to vector (s) susceptibility [7, 12], it is crucial to investigate the efficacy of selected malaria vector control interventions towards the control of *An. stephensi*, especially in areas where research has not yet covered in a holistic manner.

## Methods

### *Anopheles stephensi* larval and pupal collection sites

**Fig. 1:**
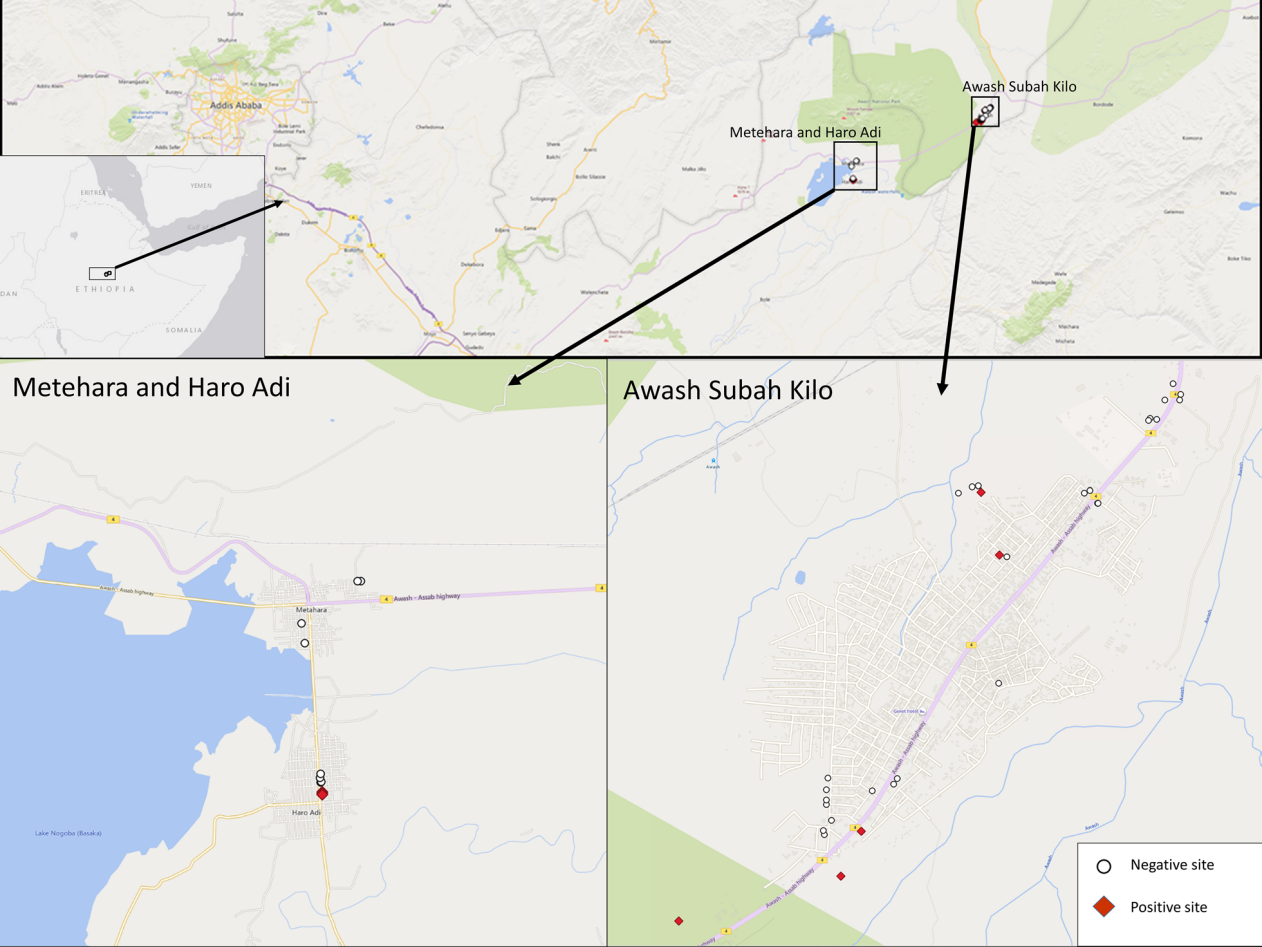
Map of *Anopheles stephensi* larvae and pupae collection habitats

Larvae and pupae of *An. stephensi* were collected from Awash Subah Kilo Town (also spelled as Awash Sebat Kilo in other publications) and Haro Adi around Metehara from January 2021 to June 2021. Awash Subah Kilo Town is located in Administrative Zone 3 of the Afar Regional State, just above a gorge of the Awash River, after which it is named. The town lies on the Addis Ababa–Djibouti Railway line at about 217 km from Addis Ababa. This town is the largest settlement in Awash Fentale district, lying at a longitude of 08°59′N 40°10′E at an elevation of 986 m. Metehara is also a town in central Ethiopia; located in the East Shewa Zone of the Oromia Regional State, on a longitude of 08°54′N 39°55′E, at an elevation of 947 m above sea level. Haro Adi village, from where the larvae and pupae of *An. stephensi* were collected, is a village to the south of Metehara Town along Lake Beseka located about two kilometers away from Metehara Town (Table 1 and Fig. 1).

**Table 1:** Description of larvae and pupae positive habitats visited from February 2021 to June 2021

Site	Geographic location				Region	Climate zone	*Anopheles* species
Awash Subah Kilo	Habitat type	Latitude	Longitude	Altitude	Afar	Semi-arid	*An. stephensi*
Habitat 1	Cistern	8^◦^58′59.88″N	40^◦^9′39.24″E	944 m	“	“	“
Habitat 2	“	9^◦^0′5.32″N	40^◦^10′2.64″E	807 m	“	“	“
Habitat 3	“	8^◦^59′53.23″N	40^◦^10′6.24″E	934 m	"	“	“
Habitat 4	“	8^◦^58′42.52″N	40^◦^9′3.6″E	940 m	"	“	“
Habitat 5	“	8^◦^58′51.24″N	40^◦^9′9.58″E	938 m	“	“	“

Haro Adi (Metehara)	Habitat type	Latitude	Longitude	Altitude	Region	Climatic zone	*Anopheles species*
Habitat1	Cistern	8^◦^52′20.28″N	39^◦^55′11.64″E	964 m	Oromia	Semi-arid to dry sub-humid	*An. stephensi*
Habitat2	“	8^◦^52′19.56″N	39^◦^55′11.64″E	968 m	“	“	“
Habitat3	“	8^◦^52ʹ18.84″N	39^◦^55ʹ11.64″E	967 m	“	“	“

^*^See Additional file 1 for complete list of sites including habitats negative for *An. Stephensi*

A total of 45 breeding sites/habitats, in and around the towns of Awash Subah Kilo and Metehara and Haro Adi village areas were visited for larval and pupal surveys. Of these, 31 breeding habitats were from Awash Subah Kilo Town, 7 from Metehara Town*,* and 7 from Haro Adi village. The survey of *An. stephensi* larvae and pupae was carried out in three sites, namely; *Awash* Subah Kilo Town, Metehara Town and Haro Adi around Metehara Town (Fig. 1). The survey for *An. stephensi* larvae and pupae was conducted in metal tanks near houses under construction, in jerry cans where water is reserved for daily household consumption, on cemented water banks for daily household consumption, on water reservoirs with domestic plastic (near *Metehara health cent*re), overhead water tanks, and in cemented burrows of water reserved for production of cement blocks. These sites were selected based on the previous reports of the presence of *An. stephensi* [4, 6]. The sampling of breeding sites was conducted based on the WHO guidelines for laboratory and field testing of mosquito larvicides [13]. All natural and man-made breeding sites around the study areas were assessed for the presence or absence of *An. stephensi* larvae.

### *Anopheles stephensi* larval and pupal collection

Larvae and pupae were collected using a World Health Organization (WHO) standard dipper and transferred into a plastic jar of five-litre capacity with a handle and a cover with plenty of holes to allow air circulation. The jar was used for handling and transporting the larvae and pupae. The scooped larvae and pupae were filtered using clean cheesecloth prepared for this purpose and transferred to a plastic jar. Then, approximately 1–1.5 L of water along with plant debris from the natural breeding sites was added to the jar for larvae to feed on until they reached the insectary facility.

### Rearing *Anopheles stephensi* mosquitoes

All larval instars and pupae collected from the field were transported to and subsequently reared to adults in a bio-contained insectary facility at the Aklilu Lemma Institute of Pathobiology (ALIPB). The insectary has two secured doors, with a double door at the entrance and each separate unit of the insectary has its own door and sealed glass windows, which prevent mosquitoes from escaping. During mosquito rearing laboratory conditions, such as maintaining the temperature at 27 ± 2 °C and 75 ± 10% relative humidity, were met and monitored. Upon arrival in the insectary, larvae were transferred into a white enamel plastic tray. Once larvae were removed from their natural water source in the plastic container using plastic of 1 ml micropipettes, a diet of baker’s yeast was added to the larval tray. After 5 min the tray was swirled to distribute the powder and prevent suffocation from undiluted/accumulated powder [14]. Larvae were provided with food twice per day, and trays were checked to see if food remained unconsumed. If food remained unconsumed, no additional food was added.

Sorting pupae from larvae was undertaken on a daily basis. Pupae were picked with plastic pipettes and transferred into a beaker with fresh deionized water and then transferred to adult holding cages. Adults in the cage were provided with sugar solution using soaked/wetted cotton ball placed on the top of the meshed cage. The cotton was maintained wet so that mosquitoes could feed on the sugar. The cotton balls were changed every 5 or 6 days, in order to avoid the growth of mold spores and/or fungus on the pad exposed to sugar [14]. Concurrent with sugar feeding, 3–7 days old female mosquitoes were fed on rabbit blood meals twice per week (ethical approval was obtained from Addis Ababa University-Aklilu Lemma Institute of Pathobiology (AAU-ALIPB) Ethical Review Board)). Water filled petri-dish and/or wet filter paper supported with cotton and placed on a petri dish were provided for mosquitoes to lay eggs on. Breeding of wild-collected *An. stephensi* colonies continued until the end of the study. The tests were done on F_0_, F_1_ and F_2_ generations of the field-collected larvae and pupae.

### *Anopheles stephensi* species identification

Mosquito species identification was undertaken morphologically under a dissecting microscope. Before commencing any efficacy test of the selected larvicides against *An. stephensi*, 30 adult female mosquitoes were randomly aspirated from cages. Then these mosquitoes were transferred into a glass tube and exposed to chloroform by a cotton ball wetted at the tip. Each of these mosquitoes was laid under a Olympus SZ stereomicroscope at 40X for morphological identification using the updated key to the females of Afro-tropical *Anopheles* mosquitoes, which includes *An. stephensi* [15]. All were confirmed to be *An. stephensi*. Fewer *Culex* and *Aedes* larvae were collected compared to *An. stephensi* from the same habitats. Though there were a few *Culex* and *Aedes* species larvae collected with *An. stephensi*, all emerged adults aspirated from the cage were *An. stephensi.* Typical features of the morphology of *An. stephensi* are (i) the appearance with 3 pale bands in the palpus and the two apical pale bands are very broad with speckling on palpus segment 3 and (ii) in the 2nd main dark area on the vein 1of its wing, there are 2 pale interruptions [15].

### Efficacy of *Bacillus thuringiensis* var. israelensis and temephos against *An. stephensi* larvae

*Bacillus thuringiensis* var. israelensis *(Bti);* FourStar®Briquets of a solid form; produced by *DBA FourStar Microbials* LLC. 1501 East Woodfield Road, #200W (https://www.centralmosquetocontrol.com/all-products/fourstar/fourstar-briquet-180) in January 2019 and with expiry date of December 2023, were acquired from ICIPE/ILRI. The powder form of this bacterial larvicide was weighed on digital weighing scale and prepared in increasing doses of 0.05 g, 0.1 g and 0.2 g, in such a way that it was to be applied in a container of 2000cm^2^ with one litre water volume until the dose mortality response was reached. Based on this design, first, the lowest prepared concentration of *Bti* (0.05 g/l), was added to deionized water and kept for 48 h by covering the container to prevent insects from landing or laying egg in it [16]. In order to ensure no insects entered into the larvicide-treated water, the tray remained closed. The subsequent tests were conducted following the same procedure.

In preparation to expose the larvae to larvicides, late third to early fourth instar larvae were sorted into disposable cups containing water using pipettes. Larvae were filtered first through cheesecloth over a separate container for this purpose. The filtered larvae were immediately transferred into plastic containers with an area of 2000cm^2^ and containing one litre of deionized water treated with *Bti* of 0.05 g, as per the application recommended for spot spray [17]. Batches of 25 larvae were exposed per testing container. Simultaneously, an equal number of larvae (negative controls) were tested using untreated deionized water with same number of larvae per container. The tests were conducted in four replicates. The experiment was repeated three times on different days and repeated for larvae collected from each site. Only, the lowest prepared concentration of *Bti* (0.05 g) was tested as a result of vector’s larvae susceptibility response to the larvicide.

Temephos, an emulsifiable liquid concentrate containing 500 g of active ingredient per liter, brand name BASF-Abate^®^500E, developed in Malaysia in 2018 (https://www.mkhardware.com.my/pages/pages_id/13613/) was acquired from the Ethiopian public health institute (EPHI), and tested against *An. stephensi* larvae. Following the same procedure used for *Bti* testing, temephos of 0.25 ml/l, 0.5 ml/l and 1 ml/l was prepared in increasing concentrations, until the dose response was saturated. Temephos (0.25 ml) was added to a container of 2000cm^2^ with one litre of deionized water volume using 1000 ml capacity micropipette. Four replicates were set up for each concentration and each was run three times on different days. An equal number of negative controls were set up simultaneously with deionized water. The late third and early fourth stage larvae, collected from the field and from reared adults (F_0_, F_1_ and F_2_), were used for the larvicidal test. Larvae were first collected from the tray using pipettes into disposable plastic cups containing water. Then 25 larvae were filtered and immediately transferred into the container of 1000 ml deionized water treated with 0.25 ml of temephos.

While conducting the efficacy tests of both larvicides, larval mortality was recorded after 24 h [15, 17, 18]. Same to *Bti*, only the lower prepared concentration of temephos (0.25 ml) was tested, as a result of larvae susceptibility response. Larvae that sank down to the bottom of water, in the case of temephos, and appeared floating on the water with swollen and blackened bodies, in the case of *Bti*, were considered dead. The WHO guidelines for laboratory and field testing of larvicides, states that the test should be rejected if the control mortality is > 20% or pupation is > 10% [13].

### Data analysis

The data were recorded using the WHO larvicide efficacy evaluation result recording form [13]. The data from all replicates were pooled and entered into an excel spreadsheet for analysis using STATA version 14.0.

If the control mortality was between 5 and 20%, the mortalities of treated groups were corrected according to Abbott’s formula. Tests with control mortality greater than 20% or pupation greater than 10% were discarded.

The mortality of the test sample was calculated by summing the number of dead larvae across all exposure replicates expressed as a percentage of the total number of exposed larvae.

### Ethical considerations

This study involved no human subjects and it was implemented after obtaining ethical clearance (Ref. No.: ALIPB IRB/40/2013/21, dated: Feb 10, 2021) from the IRB of Aklilu Lemma Institute of Pathobiology, Addis Ababa University.

## Results

### Collection of *Anopheles stephensi* larvae and pupae

Larvae and pupae were found only in two of the surveyed sites (Awash Subah Kilo Town and Haro Adi village around Metehara), and only in water reserved for the production of cement blocks in small manual factories for construction purposes. Larvae and pupae of *Anopheles* species were found in cement concrete water reservoirs and cisterns, while *Culex/Aedes* larvae were observed in plastic tankers, overhead metal tankers, barrels, jerry cans, domestic water reservoir plastics, burrow and some cisterns. Of the 45 total surveyed habitats, 5 out of 31 (16.2%) in Awash Subah Kilo Town, 3 out of 7 (42.9%) in Haro Adi, and none of the 7 in Metehara Town were found to be positive for *An. stephensi*. The map of *An. stephensi* larvae and pupae collection sites is visualized in Fig. 1.

### Additional reading regarding positive and negative breeding habitats

For the detailed descriptions of breeding habitats and timeliness on visit of habitats for larval and pupae collections in the study sites refer to Table 1 and Supplement 1. Habitats represented as 17–26 in Additional file 1 were visited during March 28–30/2021 and all were found negative for *An. stephensi* larvae, but *Culex/Aedes* species were found. Habitat 1 in Awash Subah Kilo Town and habitats 1 and 2 in Haro Adi village of Table 1 were found positive for larvae of *An. stephensi*, and all surveyed habitats in Metehara Town and habitats represented as 1–16 in Awash Subah Kilo Town were negative for larvae of *An. stephensi* during 2–3 February 2021. During 1–3 March of 2021, in Awash Subah Kilo Town habitat 1 in Table 1 and all surveyed habitats in Metehara Town and in Haro Adi village habitats 1–4 in Additional file 1 were negative for larvae of *An. stephensi*, whereas habitats 4 and 5 in Awash Subah Kilo Town and habitat 3 in Haro Adi village, as presented in Table 1 were positive for larvae of *An. stephensi.* During 8–10 of April 2021, habitats represented as 1–3 both in Awash Subah Kilo Town and in Haro Adi village, Table 1, were found positive for larvae of *An. stephensi.* On June 3, 2021, three sites (all cisterns) were found positive for *An. stephensi* in Awash Subah Kilo Town, and three sites (all cisterns) were found positive in Haro Adi village.

### Efficacy of *Bacillus thuringiensis* var. israelensis against *Anopheles stephensi* larvae

Out of the total of 600 exposed larvae, only one survived this bacterial larvicide after 24 h. All other larvae exposed in each replicate died within 24 h, and all larvae appeared floating on the water with swollen and blackened bodies. All 600 larvae under negative control conditions survived during the course of the experiment (24 h). The two higher doses (0.1 g and 0.2 g *Bti*) were not tested because larvae had already responded to the lowest dose of *Bti* (0.05 g).

Generally, *Bti* caused mortality of 100% and 99.7% in larvae from Awash Subah Kilo and Haro Adi around Metehara Town, respectively (Table 2).

**Table 2:** *Anopheles stephensi* larval mortality after 24 h of exposure to *Bti,* collected from Awash Subah Kilo and Haro Adi around Metehara Towns, January–March, 2021

Site	Larvicide	Concentration	No. exposed larvae	Mortality (%)
Awash	*Bti*	0.05 g/l	300	99.7
	Control(water)	Deionized water	300	0
Haro Adi	*Bti*	0.05 g/l	300	100
	Control(water)	Deionized water	300	0

Generally, *Bti* caused mortality of 100% and 99.7% in larvae from Awash Subah Kilo and Haro Adi around Metehara Town, respectively

### Efficacy of temephos against *Anopheles stephensi* larvae

All exposed larvae from both sites were susceptible to temephos and sank down to the bottom of the water within a short period of time (starting at 2 h post exposure). All of the 600 larvae exposed to 0.25 ml/l concentration of temephos were found dead within 24 h (Table 3). The two higher doses (0.5 ml and 1 ml of temephos) were not tested because larvae had already responded to the lowest doses of temephos (0.25 ml).

**Table 3:** *Anopheles stephensi* larval mortality after 24 h of exposure to temephos*,* collected from Awash Subah Kilo and Haro Adi around Metehara Towns, January–March, 2021

Site	Larvicide	Concentration	No. exposed larvae	Mortality (%)
Awash	Temephos	0.25 ml/l	300	100
	Control(water)	Deionized water	300	0
Haro Adi	Temephos	0.25 ml/l	300	100
	Control(water)	Deionized water	300	0.33

Statistical analysis was not done rather only descriptive statistics were used because nearly all control larvae survived and nearly all treated larvae died. From the total of 600 larvae in the control group, only 1 (0.2%) died and the test was accepted, without correction.

## Discussion

In this study, larvicide bioassays revealed that larvae of *An. stephensi* from two study localities in Ethiopia *(*Awash Subah Kilo and Haro Adi around Metehara Towns) were susceptible to both *Bti* powder and temephos liquid formulation at the lowest prepared doses. The findings also suggested that there is no difference in susceptibility status to the tested larvicides between larvae collected from the two sites.

The specimens were not stored for further molecular confirmation because of financial limitations and the inability to preserve the specimens for a longer time. However, rearing of the colony in the insectary has continued. F_0_, F_1_ and F_2_ larvae were also used to avoid shortage of test mosquitoes and also for confirmation of their species.

The bacterial larvicide, *Bti*, was efficient in killing 99.7% of exposed *An. stephensi* larvae, at the concentration of 0.05gm/l water. This finding is inline with studies conducted in Iran, though tested with different concentration units of 512 and 4096 ppm for Bio- flash® granules and powder formulation after 24 h post-treatment, that *An. stephensi* larvae were seen 100% susceptible [20]. The finding of this study, aligns with the laboratory test findings on 0.046 mg/L and 0.149 mg/L, and 0.05, 0.1, 0.2, 0.5, and 1 g/m^2^ dosages of *Bti* against *An. stephensi* in Pakistan [21] and in India [22], respectively, that have shown high efficacy against *An. stephensi* larvae within 24 h of post treatment.

A field based study conducted in western highlands in Kenya [23], has also revealed that larvae of *Anopheles gambae* complex and *An. funestus* mosquitoes decreased from 7.56 larvae per dip during pre-intervention period to 3.09 post application of *Bti* FourStar^®^ and 20 week follow up. A *Bti* susceptibility study conducted against *Aedes aegypti* in Brazil [19], using laboratory colony and *Bti* powder IPS82 as a reference to field populations, has shown that all *Ae. aegypti* populations were susceptible to *Bti*. The review of 39 studies conducted on *Bti* and/or *BS* across sub-Saharan African countries in laboratory and semi-field conditions against *Anopheles gambiae* complex and *An.funestus* have also revealed that these larvicides were effective in reducting larval density, vector density, vector biting and malaria transmission in most of the tested areas [18]. The findings of these studies, though with different methodological approach and on different mosquitoes species, strengthen the result of this study. Future work could consider testing the lower concentrations of *Bti* against larvae. The residual efficacy of the larvicides was not included in this study.

In this study, temephos showed 100% efficacy in killing all exposed *An. stephensi* larvae. This finding is similar to findings from other countries and other studies within Ethiopia [7]. Laboratory based studies in India and southern Iran revealed that larval bioassays on *An. stephensi* collected from the field were susceptible to a temephos larvicide diagnostic dose of (0.25 mg/l) [24, 25]. The only larval habitats found to be positive for *An. stephensi* were permanent water containers filled with water for the purpose of cement block production during January through March 2021 larval collections. Unlike findings by others [6–8], barrels, domestic plastic water reservoirs, overhead water containers, and Jerry Cans were negative (for unexplained reason) for larvae and pupae of *An. stephensi* in Awash Subah Kilo, Metehara and Haro Adi, around Metehara Town.

## Conclusion

The present study revealed that *An. stephensi* larvae from two locations, Haro Adi around Metehara Town and Awash Subah Kilo Town, are susceptible to *Bti* and temephos larvicides. Both *Bti* and temephos were found to be 99.7% and 100% effective in killing *An*. *stephensi* larvae, respectively. The preference of this vector for breeding in artificial habitats suggests possible control through the application of larvicides to these fixed habitats. Further laboratory and field-based studies are necessary to determine efficacy of larvicides against *An. stephensi* and other malaria vectors at different localities and presumably under field settings. Exhaustive assessment of breeding sites and identifying the cohabitants of this vector can also help in identifying effective tool(s) to control these vectors in an integrated approach.

### Supplementary Information

**Additional file 1: **Description of larvae and pupae negative breeding habitats visited during 2–3 Feb.2021, 1–3 March 2021 and 28–30 March 2021.

## Data Availability

All datasets on which the conclusions of this study relied on are presented in this paper.

## Abbreviations

AAU: Addis Ababa University
ALIPB: Aklilu Lemma institute of pathobiology
BS: *Bacillus sphaericus*
BASF: Baden aniline and soda factory
*Bti*: *Bacillus thuringiensis* Var. israelensis
CG: Corn granule
EPHI: Ethiopian public health institute
GPS: Global positioning system
ICIPE: International center of insect physiology and ecology
ILRI: International livestock research institute
IRB: Institutional review board
IRS: Indoor residual spraying
ITU: International toxic units
IVM: Integrated vector management
LLINs: Long lasting insecticide impregnated Nets
LSM: Larval source management
MOH: Ministry of health
RH: Relative humidity
WDG: Water dispersible granule
WHO: World Health Organization
