# Correction: Laboratory-based efficacy evaluation of *Bacillus thuringiensis* var. israelensis and temephos larvicides against larvae of *Anopheles stephensi* in Ethiopia

**DOI:** 10.1186/s12936-023-04730-z

**Published:** 2023-10-17

**Authors:** Abebe Teshome, Berhanu Erko, Lemu Golassa, Gedeon Yohannes, Seth R. Irish, Sarah Zohdy, Sisay Dugassa

**Affiliations:** 1 National Malaria Elimination Programme, Ministry of Health Ethiopia, PO Box 1234, Addis Ababa, Ethiopia; 2 Aklilu Lemma Institute of Pathobiology, Addis Ababa University, PO Box 1176, Addis Ababa, Ethiopia; 3 Department of Zoological Sciences, Addis Ababa University, PO Box 1176, Addis Ababa, Ethiopia; 4 Swiss Tropical and Public Health Institute (Swiss TPH), 4123 Allschwil, Switzerland; 5 Centers for Disease Control and Prevention, US President’s Malaria Initiative, Atlanta, GA USA

**Correction: Malaria Journal (2023) 22:48** **https://doi.org/10.1186/s12936-023-04475-9**

Following publication of the original article [1], the authors flagged the following errors: in the subsection ‘Efficacy of Bacillus thuringiensis var. israelensis and temephos against *An. stephensi* larvae’, they had referred to VectoBac WDG instead of FourStar^®^Briquets; in the Discussion and the Conclusion, they had referred to ‘Bti VectoBac’ where it should just say ‘Bti’. The authors thank you for reading and apologize for any inconvenience caused.
