# Supplementary material for: Laboratory-based efficacy evaluation of Bacillus thuringiensis var. israelensis and temephos larvicides against larvae of Anopheles stephensi in ethiopia
Source: Malar J. 2023 Feb 9;22:48. doi: 10.1186/s12936-023-04475-9 (PMC9912598; doi:10.1186/s12936-023-04475-9)
Supplement: Supplementary file 1 — Additional file 1: Description of larvae and pupae negative breeding habitats visited during 2–3 Feb.2021, 1–3 March 2021 and 28–30 March 2021. [file 12936_2023_4475_MOESM1_ESM.doc]

**Supplement 1.** Description of larvae and pupae negative breeding habitats

| **Site** | **Geographical Location** | | | | **Region** | **Climate zone** | ***Anopheles species*** |
| --- | --- | --- | --- | --- | --- | --- | --- |
| **Awash Subah Kilo** | **Habitat type** | **Latitude** | **Longitude** | **Altitude** | **Afar** | **Semi-arid** | **No. *An. stephensi*** |
| Habitat 1 | Metal Tanker | 9^◦^0'3"N | 40^◦^10'25.42"E | 908 | Afar | Semi-arid | Other species* |
| Habitat 2 | Cistern | 9^◦^0'31.89"N | 40^◦^10'25.47"E | 906 | " | " | " |
| Habitat 3 | " | 9^◦^0'5.09"N | 40^◦^10'22.79"E | 900 | " | " | " |
| Habitat 4 | " | 9^◦^0'5.61"N | 40^◦^10'23.87"E | 902 | " | " | " |
| Habitat 5 | Barrel | 9^◦^0'26.18"N | 40^◦^10'40.17"E | 893 | " | " | " |
| Habitat 6 | Jerry Can | 9^◦^0'24"N | 40^◦^10'41.68"E | 902 | " | " | " |
| Habitat 7 | Barrel | 9^◦^0'23.06"N | 40^◦^10'41.4"E | 896 | " | " | " |
| Habitat 8 | Metal Tanker | 9^◦^0'23.05"N | 40^◦^10'38.51"E | 895 | " | " | " |
| Habitat 9 | Cistern | 9^◦^0'19.27"N | 40^◦^10'36.91"E | 897 | " | " | " |
| Habitat 10 | Cistern | 9^◦^0'19.5"N | 40^◦^10'35.65"E | 899 | " | " | " |
| Habitat 11 | Roto | 9^◦^0'19.06"N | 40^◦^10'35.4"E | 898 | " | " | " |
| Habitat 12 | " | 8^◦^59'52.87"N | 40^◦^10'7.69"E | 913 | " | " | " |
| Habitat 13 | Jerry Can | 8^◦^59'28.42"N | 40^◦^10'6.1"E | 905 | " | " | " |
| Habitat 14 | Metal Tanker | 9^◦^0'5.09"N | 40^◦^9'58.25"E | 895 | " | " | " |
| Habitat 15 | " | 9^◦^0'6.35"N | 40^◦^10'0.9"E | 895 | " | " | " |
| Habitat 16 | " | 9^◦^0'6.52"N | 40^◦^10'2.1"E | 899 | " | " | " |
| Habitat 17 | Cistern | 8^◦^58'59.21"N | 40^◦^9'32.06"E | 925 | " | " | " |
| Site 18 | " | 8^◦^59'0.1"N | 40^◦^9'31.8"E | 920 | " | " | " |
| Habitat 19 | " | 8^◦^59'1.98"N | 40^◦^9'33.41"E | 919 | " | " | " |
| Habitat 20 | " | 8^◦^59'5.94"N | 40^◦^9'32.41"E | 917 | Afar | Semi-arid | Other species |
| Habitat 21 | Barrel | 8^◦^59'5.95"N | 40^◦^9'32.38"E | 920 | " | " | " |
| Habitat 22 | Cistern | 8^◦^59'7.89"N | 40^◦^9'32.44"E | 921 | " | " | " |
| Habitat 23 | " | 8^◦^59'10.1"N | 40^◦^9'32.69"E | 921 | " | " | " |
| Habitat 24 | " | 8^◦^59'7.74"N | 40^◦^9'41.36"E | 924 | " | " | " |
| Habitat 25 | " | 8^◦^59'8.95"N | 40^◦^9'45.58"E | 924 | " | " | " |
| Habitat 26 | Domestic plastic | 8^◦^59'10.1"N | 40^◦^9'46.23"E | 923 | " | " | " |
| **Metehara** | **Habitat type** | **Latitude** | **Longitude** | **Altitude** | **Region** | **Climatic zone** | ***Anopheles species*** |
| Habitat 1 | Domestic plastic | 8^◦^54'0.44"N | 39^◦^55'35.1"E | 961 | Oromia | Semi-arid to dry sub-humid | Other species |
| Habitat 2 | " | 8^◦^54'26.06"N | 39^◦^55'33.11"E | 952 | " | " | " |
| Habitat 3 | Cistern | 8^◦^53'51.4"N | 39^◦^55'7.05"E | 946 | " | " | " |
| Habitat 4 | " | 8^◦^53'54.84"N | 39^◦^55'3.76"E | 925 | " | " | " |
| Habitat 5 | Domestic plastic | 8^◦^53'57.02"N | 39^◦^55'1.16"E | 942 | " | " | " |
| Habitat 6 | Cistern | 8^◦^54'1.11"N | 39^◦^55'59.01"E | 946 | " | " | " |
| Habitat 7 | " | 8^◦^53'49.3"N | 39^◦^55'1.2"E | 944 | " | " | " |
| **Haro Adi** | **Habitat type** | **Latitude** | **Longitude** | **Altitude** | **Region** | **Climatic zone** | ***Anopheles species*** |
| Habitat 1 | Domestic plastic | 8^◦^52'26.26"N | 39^◦^55'10.58"E | 961 | Oromia | Semi-arid to dry sub-humid | Other species |
| Habitat 2 | " | 8^◦^52'26.35"N | 39^◦^55'10.96"E | 950 | " | " | " |
| Habitat 3 | Cistern | 8^◦^52'28.85"N | 39^◦^55'10.2"E | 958 | " | " | " |
| Habitat 4 | Metal Tanker | 8^◦^52'31.35"N | 39^◦^55'10.84"E | 946 | " | " | " |
| *Other species is representing *Culex* and/or *Aedes* species of mosquitoes | | | | | | | |
